# Enantiomeric switching of chiral metamaterial for terahertz polarization modulation employing vertically deformable MEMS spirals

**DOI:** 10.1038/ncomms9422

**Published:** 2015-10-01

**Authors:** Tetsuo Kan, Akihiro Isozaki, Natsuki Kanda, Natsuki Nemoto, Kuniaki Konishi, Hidetoshi Takahashi, Makoto Kuwata-Gonokami, Kiyoshi Matsumoto, Isao Shimoyama

**Affiliations:** 1 Department of Mechano-Informatics, The University of Tokyo, 7-3-1 Hongo, Bunkyo-ku, Tokyo 113-8656, Japan; 2 IRT Research Initiative, The University of Tokyo, 7-3-1 Hongo, Bunkyo-ku, Tokyo 113-8656, Japan; 3 Extreme Photonics Research Group, RIKEN Center for Advanced Photonics, 2-1 Hirosawa, Wako 351-0198, Japan; 4 Photon Science Center, The University of Tokyo, 7-3-1 Hongo, Bunkyo-ku, Tokyo 113-8656, Japan; 5 Department of Physics, The University of Tokyo, 7-3-1 Hongo, Bunkyo-ku, Tokyo 113-8656, Japan; 6 Institute for Photon Science and Technology, The University of Tokyo, 7-3-1 Hongo, Bunkyo-ku, Tokyo 113-8656, Japan

## Abstract

Active modulation of the polarization states of terahertz light is indispensable for polarization-sensitive spectroscopy, having important applications such as non-contact Hall measurements, vibrational circular dichroism measurements and anisotropy imaging. In the terahertz region, the lack of a polarization modulator similar to a photoelastic modulator in the visible range hampers expansion of such spectroscopy. A terahertz chiral metamaterial has a huge optical activity unavailable in nature; nevertheless, its modulation is still challenging. Here we demonstrate a handedness-switchable chiral metamaterial for polarization modulation employing vertically deformable Micro Electro Mechanical Systems. Vertical deformation of a planar spiral by a pneumatic force creates a three-dimensional spiral. Enantiomeric switching is realized by selecting the deformation direction, where the polarity of the optical activity is altered while maintaining the spectral shape. A polarization rotation as high as 28° is experimentally observed, thus providing a practical and compact polarization modulator for the terahertz range.

## Abstract (web summary)

Spectroscopy in the terahertz range requires polarization modulation. Here, Kan *et al.* demonstrate a chirality switchable metamaterial for polarization modulation employing deformable spirals. A polarization rotation as high as 28° is achieved, providing a practical and compact polarization modulator for the terahertz range.

## Graphical abstract

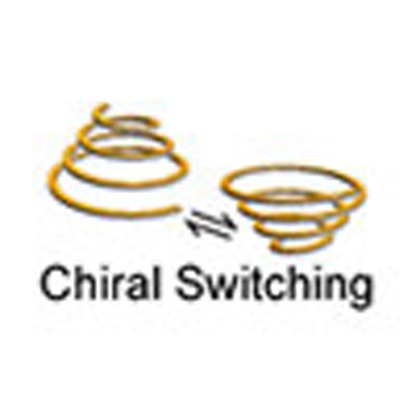

Chiral materials exhibit optical activity^1^ originating from their different responses to left and right circularly polarized light, providing a change in the polarization state of the transmitted light. These molecules take the *l*- and *d*-forms of enantiomers and exhibit the remarkable characteristic that opposite enantiomers present identical transmission spectra while reversing the polarity of the optical activity. Furthermore, some enantiomer molecules are able to switch to the other handed isomer when subjected to stimuli such as heat or light^2^,^3^. These features satisfy the requirements of a polarization modulator for controlling the polarization state without altering the light intensity. We took advantage of these features to construct a polarization modulator in the terahertz (THz) range, which is required for THz polarimetric measurements employed in the above-mentioned important applications^4^,^5^,^6^,^7^,^8^,^9^,^10^. For example, the THz frequency overlaps several molecular vibration modes^6^; therefore, vibrational circular dichroism spectroscopy can be used to determine the structures of molecules, such as the tertiary or chirality structure, without crystallization^7^,^8^,^11^. Whereas active polarization control methods in the THz frequency range have been restricted to large specialized systems^12^,^13^,^14^,^15^, the proposed device will provide a simple and practical polarization modulator that can be inserted into any part of a THz experimental setup. For instance, by simply inserting such a device into the THz imaging system with a quantum cascade laser^16^, the handedness of the incident circularly polarized THz beams can be switched synchronized with the frame rate of the imager. Real-time THz circularly polarization imaging can thus be realized.

The key issues in employing a principle equivalent to enantiomers are the realization of a large optical activity and enantiomeric switching. Because optical activity is a first-order spatial dispersion effect originating from the non-locality of the light–matter interaction^17^, achieving a large three-dimensional (3D) spatial variation in the chiral structures is essential for obtaining sufficient optical activity. In addition, the enantiomeric switching requires a mirror-image inversion of 3D structures. Here we focused on chiral metamaterials composed of artificial structures without mirror symmetry at the sub-wavelength scale because they offer optical activities larger than those of natural materials^18^,^19^,^20^,^21^,^22^,^23^. In the THz region, the active tuning of chiral metamaterials has been pursued^24^,^25^,^26^,^27^,^28^,^29^. However, these metamaterials have been primarily two dimensional and tuned by photoexcitation^24^,^25^,^28^, which is generally not suitable for enantiomeric switching. We have recently reported enantiomeric switching with photoexcitation by constructing a spatial light modulator^28^, but the polarization effect was too small for practical use because its quasi-two-dimensional configuration lacked sufficient spatial dispersion. Handedness switching with a 3D chiral metamaterial by photoexcitation has also been reported^25^, but the switched structures lack enantiomeric symmetry, resulting in asymmetric optical activity spectra. Therefore, there remains the need for a metamaterial satisfying both of these requirements; large optical activity and enantiomeric switching ability.

To satisfy these requirements, here we propose a Micro Electro Mechanical Systems (MEMS) chiral metamaterial facilitated by a deformable 3D chiral structure with the ability to switch between mirror images. The 3D spirals present large optical activity, and enantiomeric switching gives rise to the symmetrical optical activity spectral shapes between the different handedness structures. This technology provides new ways of manipulating polarization states of the THz electromagnetic waves.

## Results

### Handedness-switchable chiral metamaterial

**Figure 1:**
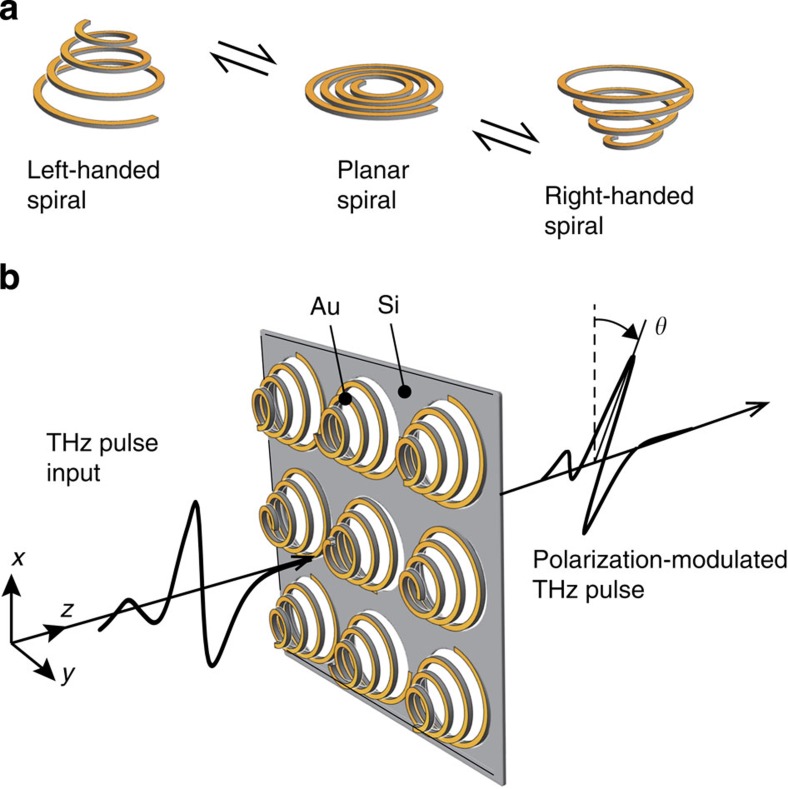
Configurations of the MEMS spiral metamaterial. (**a**) Enantiomeric chirality switching of the spiral structures is performed by changing the deformation direction of a planar Archimedean spiral. (**b**) The spiral structures are arrayed to form a chirality-switchable metamaterial. The polarization state of the THz wave is modulated by the optical activity of the metamaterial as the light passes through the metamaterial.

The working principle of the MEMS chiral metamaterial is shown in Fig. 1a. When a planar Archimedean spiral is actuated in the upward vertical direction, the spiral becomes left handed (LH) (Fig. 1a). A reversal of the direction provides a right-handed (RH) spiral, a mirror image of the LH spiral. This reversal achieves chirality switching while maintaining the enantiomeric symmetry. The spiral deformation modulates the polarization state of THz wave passing through it (Fig. 1b). To enhance the spatial dispersion, an enlargement of the 3D spatial variation, that is, a large spiral deformation, becomes necessary. Although electrostatic force has been commonly used for MEMS actuation, it is not suitable to obtain a large deformation, as we previously reported^29^. Therefore, we herein employed a unique pneumatic force actuation, which provides a significantly larger deformation than the electrostatic force (Supplementary Note 1 and Supplementary Fig. 1).

**Figure 2:**
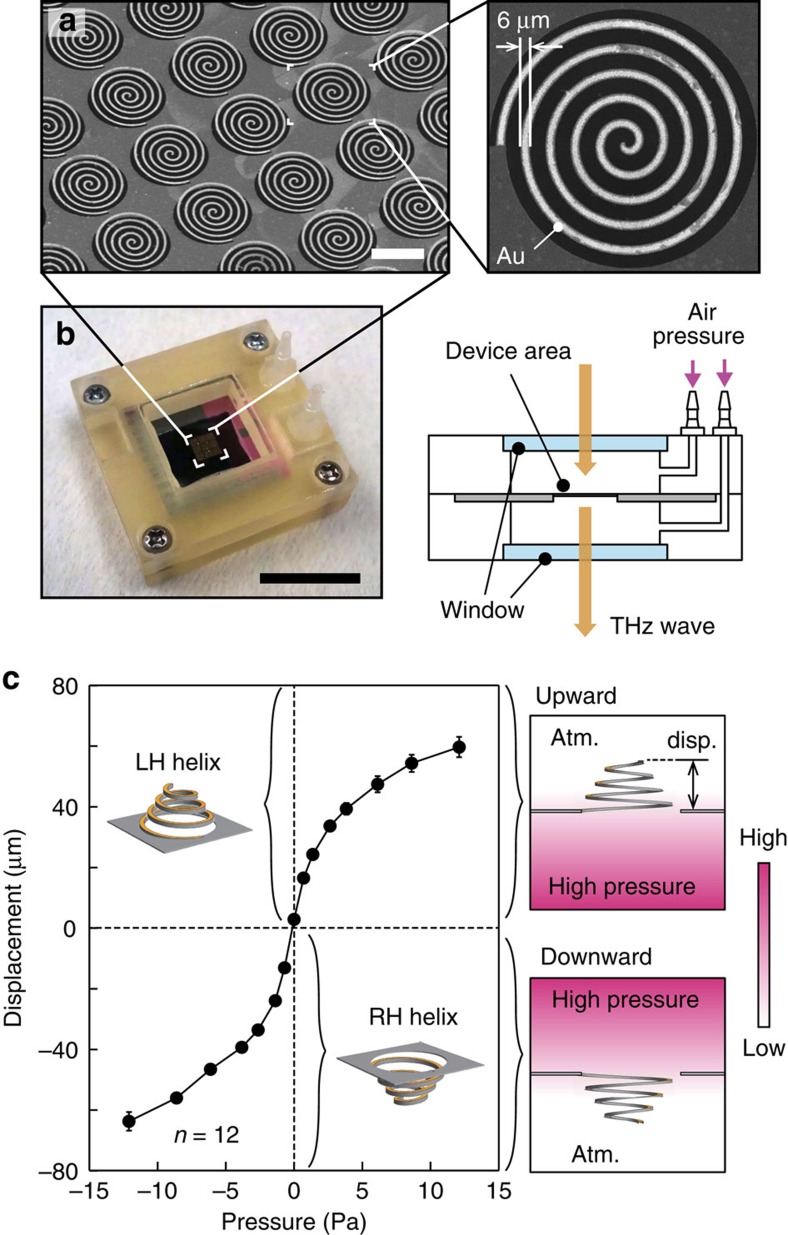
Scanning electron microscope (SEM) images and the actuation characteristics. (**a**) SEM images of the spiral metamaterial with *C*_1_ symmetry. A bar indicates 100 μm. The right-side image shows a single spiral. (**b**) A photograph and a schematic diagram of the pressure application jig and the metamaterial chip (see also the Method section). A bar in the photograph indicates 20 mm. (**c**) Displacement of the spiral structure with respect to the applied pressure. The displacement was obtained at the spiral centre with a laser 3D profiler as N_2_ pressure was applied to the spirals (see also the Methods section and Supplementary Note 1 for detailed profiles and photographs, and a movie). The sign of the pressure is positive when the pressure is applied to the bottom chamber, resulting in LH spirals, and vice versa. The plotted points correspond to the average of 12 spiral deformations with error bars of s.d. The figures located to the right of the plot indicate the manner of pressure application for the upward or downward deformation directions (Atm., and disp. correspond to atmospheric pressure, and displacement.).

Details regarding the MEMS fabrication procedures and the dimensions of the spiral metamaterial are provided in the Methods section. Observed from the Au-deposited side, the spiral turn direction was clockwise (Fig. 2a). The direction of deformation was switched by selecting whether the upper or lower chamber was supplied with N_2_ pressure (Fig. 2b). Laser microscopy analysis of the spiral profile revealed that the deformation was symmetrical with respect to the origin (Fig. 2c). The height of the spiral centre reached 60 μm in both the upward and downward directions at ±10 Pa. Laser Doppler analysis indicated that the mechanical resonance of the spiral did not appear until ∼3 kHz (Supplementary Note 2 and Supplementary Fig. 2), which assures that the polarization modulation can be achieved at a frequency as high as a few kHz.

### Modulation of THz optical activity

**Figure 3 f3:**
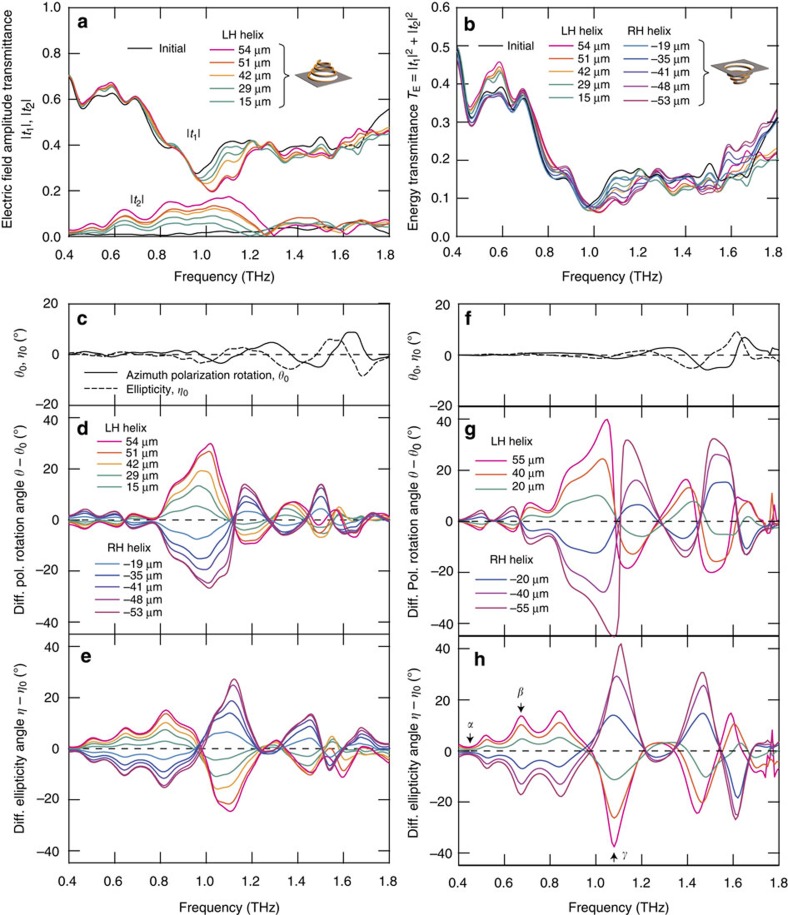
Experimental results of the proposed spiral metamaterial for different deformation conditions. (**a**) Electric field amplitude transmittance. The black lines correspond to the condition of no applied pressure, wherein finite initial inclination exists. (**b**) Energy transmittance. (**c**) Azimuth polarization rotation *θ*_0_ and ellipticity *η*_0_ angles for the condition of no applied pressure. (**d**) Differential azimuth polarization rotation angles *θ*−*θ*_0_. (**e**) Differential ellipticity angles *η*−*η*_0_. The colour legend is as in **d**. (**f**) Calculated azimuth polarization rotation *θ*_0_ and ellipticity *η*_0_ angles for the condition of no applied pressure. Modelling details are provided in the Method section. (**g**) Calculated azimuth polarization rotation angle *θ*−*θ*_0_ spectrum for six deformation conditions. (**h**) Calculated differential ellipticity angle *η*−*η*_0_ spectrum for six deformation conditions, with the same colour legend as in **g**. Diff., differential; Pol., polarization.

The optical activity modulation was investigated using THz time-domain spectroscopy (THz-TDS), as described in the Methods section and a previous report^23^. The incident THz pulses were linearly polarized along the *x* axis as shown in Fig. 1. The complex Jones matrices of the spiral metamaterials are derived from experimentally obtained data. The diagonal and off-diagonal Jones matrix components are denoted as *t*_1_ and *t*_2_, respectively (derivations are shown in the Methods section and Supplementary Note 3). 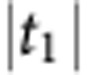 and 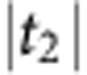 are plotted in Fig. 3a. 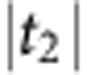 is almost zero for the initial non-pressure condition, indicating the absence of a polarization rotation. The increase in 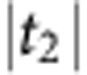 for increasing deformation indicates the emergence of optical activity. The energy transmittance 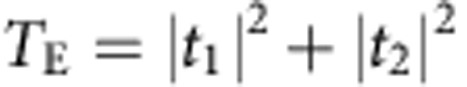 did not vary with the degree of deformation for frequencies from 0.6 to 1.5 THz (Fig. 3b).

The polarization azimuth rotation angle *θ* and ellipticity angle *η* were calculated from *t*_1_ and *t*_2_ (see equation (13) in Supplementary Note 3). Both angles for the initial non-pressure condition, denoted as *θ*_0_ and *η*_0_, are shown in Fig. 3c. The chirality of flat spirals should be almost lost because the substrate-induced effect is negligible in the THz region^23^. A small polarization change, for example, of 1.3° in the azimuth rotation was, however, observed near 1 THz for the initial non-pressure condition. This is attributed to an initial slight spiral inclination, presumably due to residual stress. The differences in the polarization spectra *θ* and *η* with respect to *θ*_0_ and *η*_0_ are plotted in Fig. 3d,e. As the deformation increased, the optical activity increased monotonically. There was a distinctive resonance in the azimuth rotation near 1.0 THz, where the ellipticity exhibited a dispersive curve and crossed zero. This concurrence indicates that the two spectra satisfy the Kramers–Kronig relations. It should be noted that the conventional definitions of *θ* and *η* are employed in this paper; these values were derived from the Stokes parameters as explained in Supplementary Note 3 in detail. The sign of either *θ* or *η* should be reversed to satisfy the Kramers–Kronig relations (see also page 62 in ref. 1). In addition, the optical activity polarities of the LH and RH spirals are opposite, with almost identical spectral shapes, thus ensuring enantiomeric handedness switching. The azimuth rotation *θ* exhibited maximum magnitude of 28.1° and −28.7° at 1.0 THz for the LH and RH spirals, respectively. The maximum magnitude of the ellipticity *η* for the LH spiral was smaller than that for the RH spiral, measuring −22.3° and 29.7°at 1.1 THz. The resonant frequency in the spectrum remained constant regardless of the spiral deformation change^30^.This behaviour enables independent control of the azimuth rotation and the ellipticity. At 1.0 THz, for example, the deformation changes the azimuth rotation, while the ellipticity is unchanged. A similar controllability holds for the 1.1 THz where the deformation only changes the ellipticity with keeping the azimuth rotation.

Simulations were performed (Fig. 3f–h), and the features of the experimental spectra were well reproduced at nearly all frequencies. Although the experimentally obtained maximum values of both the polarization azimuth rotation and ellipticity angles were smaller than those of the simulated data, this reduction in the maximum values can be largely attributed to the finite frequency resolution of the THz-TDS system. If we apply the simulation data with a convoluting operation, equivalent to the effect of the finite frequency resolution (0.08 THz) of the THz-TDS system, the processed simulation data exhibit high consistency with the experimental data (Supplementary Note 4 and Supplementary Fig. 3). This result conversely implies that the amplitude of the ellipticity may have been as large as 40° at the peak wavelength using the metamaterial. It was also numerically confirmed that the optical activity can be further enhanced by increasing the vertical deformation of the spirals, resulting in, for example, 90° polarization rotation at the resonant frequency (Supplementary Note 5 and Supplementary Fig. 4a,b). Moreover, the simulation also demonstrated that the resonant frequency can be tuned by changing the size of the spiral rather than the period of them (Supplementary Note 5 and Supplementary Fig. 4c–f).

**Figure 4:**
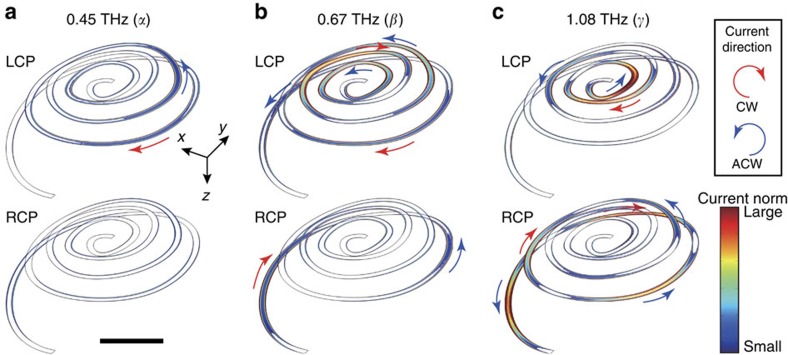
Time-averaged current norm distributions on the 55-μm-height spiral for right and left circularly polarized light (denoted as RCP and LCP, respectively). (**a**–**c**) Correspond to the distributions at the frequency of 0.45, 0.67 and 1.08 THz, respectively. The corresponding frequencies are indicated by markers in Fig. 3h as *α*, *β* and *γ*, respectively. A bar in **a** indicates 50 μm. The arrows in these distribution figures indicate the current direction at a certain time: red and blue arrows correspond to the clockwise (CW) and anti-clockwise (ACW) current directions seen from the −*z* direction to +*z* direction.

To investigate the microscopic origin of the observed polarization effect, we calculated the current norm distributions generated on the spiral for the irradiations of the circularly polarized light as shown in Fig. 4a–c. At 0.45 THz, the generated currents are weak for both types of circularly polarized light because the frequency is off-resonant. For resonant frequencies of 0.67 THz, the currents generated for left circularly polarized light appear to exceed those for right circularly polarized light. The induced current power for the left circularly polarized light was actually higher than that for the right by 4.5-fold (for the derivation of the current power, see the Methods section). The generated current is thought to produce reflection of the incident circularly polarized light^22^, and thus the transmittance of the left circularly polarized light should be smaller than that of the right circularly polarized light at 0.67 THz. These transmittance differences produce a positive ellipticity value, and thus the current distributions are consistent with the ellipticity angle spectra (see Supplementary Note 3 about the definition of ellipticity). Similar relations hold for the current distributions at 1.08 THz, at which the induced current power for the right circularly polarized light dominated by 1.4-fold, giving rise to the negative ellipticity value.

### Effect of the rotational symmetry

**Figure 5:**
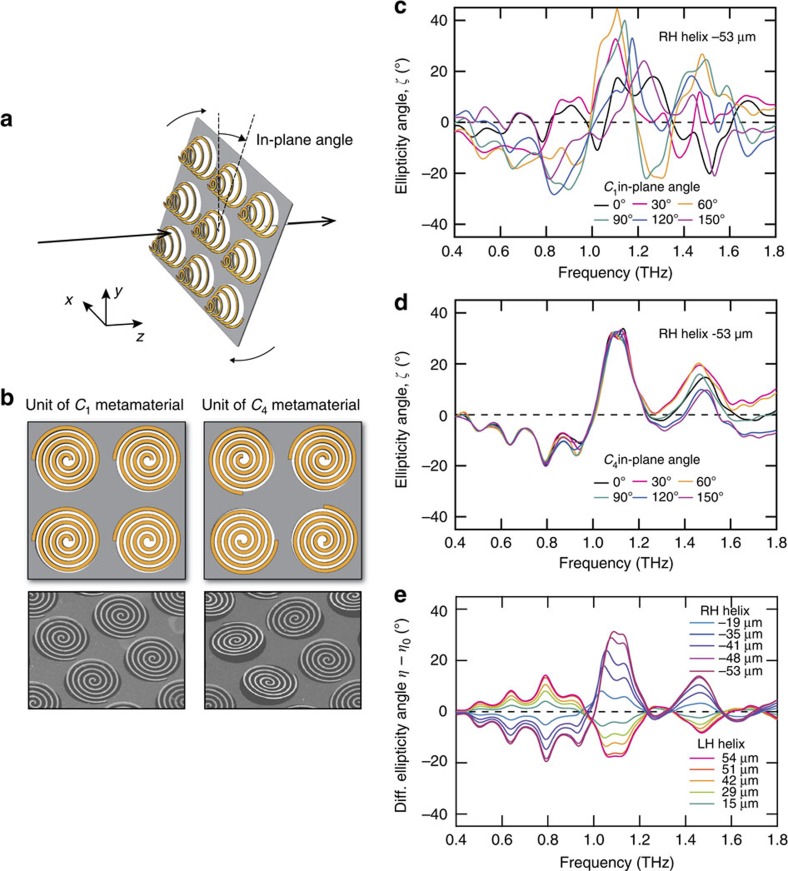
Elimination of birefringence by arraying the spirals in a configuration with *C*_4_ symmetry. (**a**) A definition of the in-plane angle. (**b**) Top views and scanning electron microscope (SEM) images of the arrangements of the *C*_1_ and *C*_4_ metamaterials. The four spirals of the *C*_4_ metamaterial unit are arranged to have differing angles of rotation around the axis normal to the device surface, with angles of 0, *π*/2, π and 3*π*/2. (**c**) Spectra of the ellipticity angle *ζ* without averaging over the in-plane angle for the *C*_1_ metamaterial for six in-plane angles. All ellipticity spectra correspond to spirals with a centre deformed height of −53 μm. For a derivation procedure, see equations in Supplementary Note 3. (**d**) Spectra of the ellipticity angle *ζ* without averaging over the in-plane angle for the *C*_4_ device for six in-plane angles; the spirals have a centre deformed height of −53 μm. (**e**) Differential (Diff.) ellipticity angles *η*−*η*_0_ of the *C*_4_ spiral metamaterial.

Although the spiral appears to have a small linear anisotropy because of its circle-like shape, its symmetry for rotation around the axis normal to the device surface belongs to the *C*_1_ group because of the existence of the spiral endpoint, giving rise to birefringence. The transmittance and polarization spectra thus differ depending on the in-plane angle (Fig. 5a). For an evaluation of the birefringence, the ellipticities *ζ* for several in-plane angles were calculated without averaging over the in-plane angle, unlike the previous experimental section (Fig. 5c). The calculation shows that the ellipticity *ζ* is strongly dependent on the in-plane angle because of the birefringence, whose amplitude is comparable to that of the optical activity. For example, at 1.1 THz, the ellipticities *ζ* fluctuate from 3.2° to 42.3° depending on the in-plane angle, whereas the ellipticity amplitude *η* resulting from the optical activity is ∼28°, as shown above. Independence of the ellipticity from the in-plane angle is important for sensitive measurements and exploitation of the optical activity. To eliminate the birefringence, we prepared another unit structure consisting of four spirals with differing angles of rotation around the axis normal to the device surface (Fig. 5b). The ellipticity of this *C*_4_ symmetrical structure is shown in Fig. 5d. In contrast to the *C*_1_ case, we observed a linearly isotropic response, independent of the in-plane angle. The differential ellipticity spectra *η−η*_0_ were almost the same as those of the *C*_1_ metamaterial (Fig. 5e). The differential polarization azimuth rotation angle spectra exhibited identical characteristics (Supplementary Note 6 and Supplementary Fig. 5). Thus, the *C*_4_ arrangement enabled polarization modulation due to only the optical activity.

## Discussion

We have demonstrated a deformable MEMS spiral metamaterial in the THz frequency range. The large optical activity resonance at 1.0 THz provided a maximum ellipticity angle as large as 28°. The directional switching of the pneumatic actuation enabled an optical activity polarity reversal while maintaining a constant energy transmittance and spectral shape, thus achieving enantiomeric handedness switching, which has been difficult to obtain in chiral metamaterials. The *C*_4_ arrangement eliminated the birefringence. Because the deformable MEMS metamaterial enabled large tunable optical activity with a compact device configuration, it can serve as a polarization modulator in the THz range. Further improvement of the amplitude of the optical activity will be possible by increasing the central height of the spiral at the deformation. We estimate that narrowing the interspace between the spiral arms and the addition of micro bristle-like structures on the spiral beam edges will increase the deformation force the spiral beam receives from the air flow^31^, enabling greater deformation of the spirals. Combined with a real-time two-dimensional imager (a THz camera)^32^, the proposed metamaterial can be applied for sensitive real-time measurement system of the circular dichroism spectra of chiral molecules such as amino acids with modulation of the video rate speed (for example, 30 Hz) in a future work. Employment of fast electromagnetic valves into pneumatic channels will be promising for increasing the actuation frequency to ∼1 kHz. For higher-frequency modulation above 1 kHz, actuation with a sonic wave may be appropriate^33^. The dynamic reconfiguration capabilities of the proposed metamaterial in combination with the unique optical characteristics of metamaterials, such as negative refractive index media^34^ and extrinsic chirality generation for oblique incidence^35^, may enable the development of novel tunable functional devices. This paper reports the first demonstration of MEMS enantiomer switching and proposes a compact and practical THz polarization modulator, leading to the realization of fundamental optical components in the emerging THz field.

## Methods

### Device fabrication and configurations

The spiral metamaterial was formed on a silicon-on-insulator wafer (top Si 300 nm/buried SiO_2_ 400 nm/handling Si 200 μm). A 45-nm-thick Au film was deposited as an electrically conductive layer by electron beam evaporation on the top Si layer of the wafer. The spiral patterns were formed in the Au film by photolithography and etching. The top Si layer within the spiral areas was then etched by reactive ion etching with the Au acting as a mask to form spiral beams. The handling Si layer was etched from the backside of the wafer to remove the handling Si beneath and between the spirals. Finally, the SiO_2_ beneath and between the spirals was removed by etching with hydrofluoric acid vapour. To inhibit sticking of the spiral beams, the wafer was heated to 40 °C to evaporate unwanted water. The metamaterials were thus fabricated on the top Si suspended membrane with an area of 5 × 5 mm. Identical spirals were arrayed with a 170-μm pitch. The Archimedean spiral is expressed by 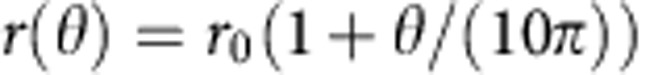, where *r* is the radius at the angular position *θ*, *θ* is the angular position of the spiral beam and *r*_0_ is the initial radius of the spiral. The spiral beam was drawn for *θ* from 0 to −10*π*, producing a clockwise-turning spiral. The number of turns for the spiral was thus 5, and the beam width of the spiral was 6 μm.

### Pneumatic actuation

Pneumatic force was used to deform the spirals. For deformations in both the upward and downward directions, a 3D-printed jig was prepared, as shown in Fig. 2b. The jig was composed of top and bottom parts, and each part had an air channel to guide the pneumatic pressure. Windows transparent at THz frequencies (Zeonex 480R, Nihon Zeon, Japan) were embedded in the jig for subsequent THz-TDS measurements. The spiral metamaterial chip was sandwiched between these two parts, and the pneumatic force was supplied through these air channels. The pressure source was a N_2_ gas cylinder regulated by a pressure injector (PV820, World Precision Instruments, USA). For both the upward and downard deformations, the side without applied pressure was maintained at atmospheric pressure. The pressure and deformation property data shown in Fig. 2c were obtained by measuring the differential pressure between the upper and lower chambers (KL17 (–50 to 50 Pa), Nagano Keiki, Japan). The pressure injector and the pneumatic jig were connected with 2-m-long silicone tubes such that the pneumatic jig could be installed in the THz-TDS setup.

### Terahertz time-domain spectroscopy

The THz-TDS measurements were performed using a Ti:sapphire regenerative amplifier (RegA 9000, Coherent Inc., USA) with a 120-kHz repetition rate, an 803-nm centre wavelength and a 120-fs pulse duration. THz pulses were generated by optical rectification in a LiNbO_3_ crystal using the tilted pulse front excitation method and were detected via electro-optic sampling using a (110)-oriented ZnTe crystal with a thickness of 1 mm. The generated THz radiation was focused to a diameter of ∼1 mm onto the sample at normal incidence by a gold-coated off-axis parabolic mirror with a 6-inch effective focal length. The polarization states of the transmitted THz waves were measured using two wire grid polarizers. Further details regarding the analysis of the polarization states are described elsewhere^23^.

### Derivation of the Jones matrix

The complex Jones matrix of the spiral metamaterial was evaluated using the THz-TDS-based polarimetry measurement. Linearly polarized THz wave pulses were introduced to the metamaterial, and the polarization states of the output THz pulses were measured. The transmission spectra of the metamaterial were measured two times in the experiment: the first measurement was obtained in the initial metamaterial position, which was defined as the 0° position around the *z* axis. For the second measurement, the metamaterial was rotated by 90° around the *z* axis from the first measurement configuration, and the transmission spectra were measured again to obtain the four Jones matrix components in equation (1), as shown below. The complex Jones matrix T in the frequency domain describes the linear response of the sample:


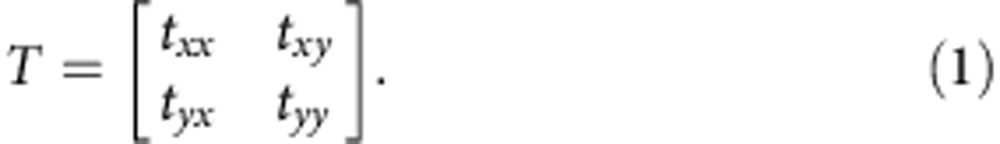


Each matrix component was experimentally determined. The optical activity is independent of the in-plane angle, which is defined as the rotation angle of the device about the normal axis (Fig. 5a). Thus, to extract the in-plane angle dependency and obtain components of the Jones matrix independent of the in-plane angle from the measured data, the Jones matrix T was averaged over the in-plane angle by numerical calculation procedures described in Supplementary Note 3. Following the procedure, the form of the matrix is reduced to T_avg_:


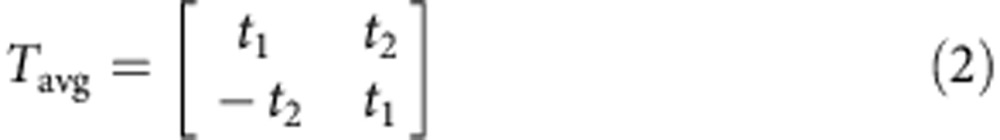


When the off-diagonal term *t*_2_ is not zero, optical activity is observed, and the polarization azimuth rotation *θ* and ellipticity *η* angles are calculated from *t*_1_ and *t*_2_. Details regarding the derivation procedure for the Jones matrix components and polarization rotations are described in Supplementary Note 3.

### Simulation model construction

A finite element calculation was performed using commercial software (COMSOL version 4.4, COMSOL, USA) to simulate the electromagnetic responses of the spiral metamaterial. A calculation model was constructed based on 3D laser scanning data. The 3D shapes of three spirals with different deformation conditions were used: a no-pressure-applied spiral, a LH spiral with a centre height of 48 μm (upward direction) and a RH spiral with a centre height of −46 μm (downward direction). For the results displayed in Fig. 3f through 3h, [Fig f3], the centre heights of the LH and RH spirals in the obtained 3D data were scaled to create simulation models with centre heights of 20, 40 and 55 μm for the LH spirals and −20, −40 and −55 μm for the RH spirals. Note that the Si layer was omitted from the calculation model, and the model was composed of only a spiral-shaped Au film in vacuum; the film was 45 nm thick and 4.7 μm wide based on SEM observations. Au is highly conductive in the THz region; thus, we set the electrical conductivity to 45.6 × 10^6^ S m^−1^, which corresponds to the value for d.c. conditions. In addition, the current distributions on the 55-μm-height LH spirals are presented for both right and left circularly polarized light incidence in Fig. 4a–c. To quantitatively compare the amount of generated current for the polarization handedness, the powers of the current for the circularly polarized light were calculated by taking a time-averaged surface integration of the square of the current density over the spiral surface.

## Additional information

**How to cite this article:** Kan, T. *et al.* Enantiomeric switching of chiral metamaterial for terahertz polarization modulation employing vertically deformable MEMS spirals. *Nat. Commun.* 6:8422 doi: 10.1038/ncomms9422 (2015).

## Supplementary Material

Supplementary Figures, Supplementary Notes and Supplementary Reference Supplementary Figures 1-5, Supplementary Notes 1-6 and Supplementary Reference

Supplementary Movie 1 A real-time movie of the MEMS spiral metamaterials actuated by the pneumatic force supplied from the bottom side
