# Supplementary material for: Enantiomeric switching of chiral metamaterial for terahertz polarization modulation employing vertically deformable MEMS spirals
Source: Nat Commun. 2015 Oct 1;6:8422. doi: 10.1038/ncomms9422 (PMC4600721; doi:10.1038/ncomms9422)
Supplement: Supplementary Figures, Supplementary Notes and Supplementary Reference — Supplementary Figures 1-5, Supplementary Notes 1-6 and Supplementary Reference [file ncomms9422-s1.pdf]

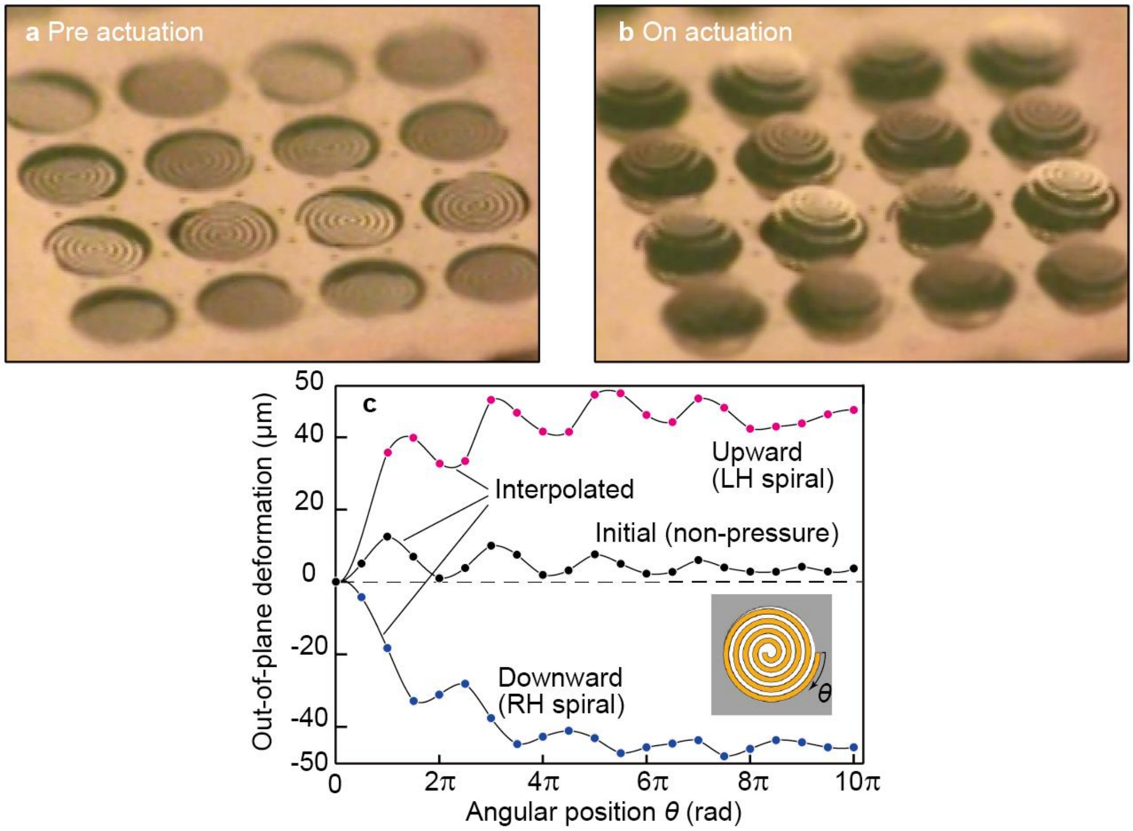

Supplementary Figure 1. Actuation characteristics of the MEMS spiral metamaterial. (a) Microscope images of the spirals in the pre-actuation and (b) on-actuation states. (c) Spiral deformation in the out-of-plane direction plotted versus angular position *θ* along the spiral beam, which is defined as shown in the inset of the plot.

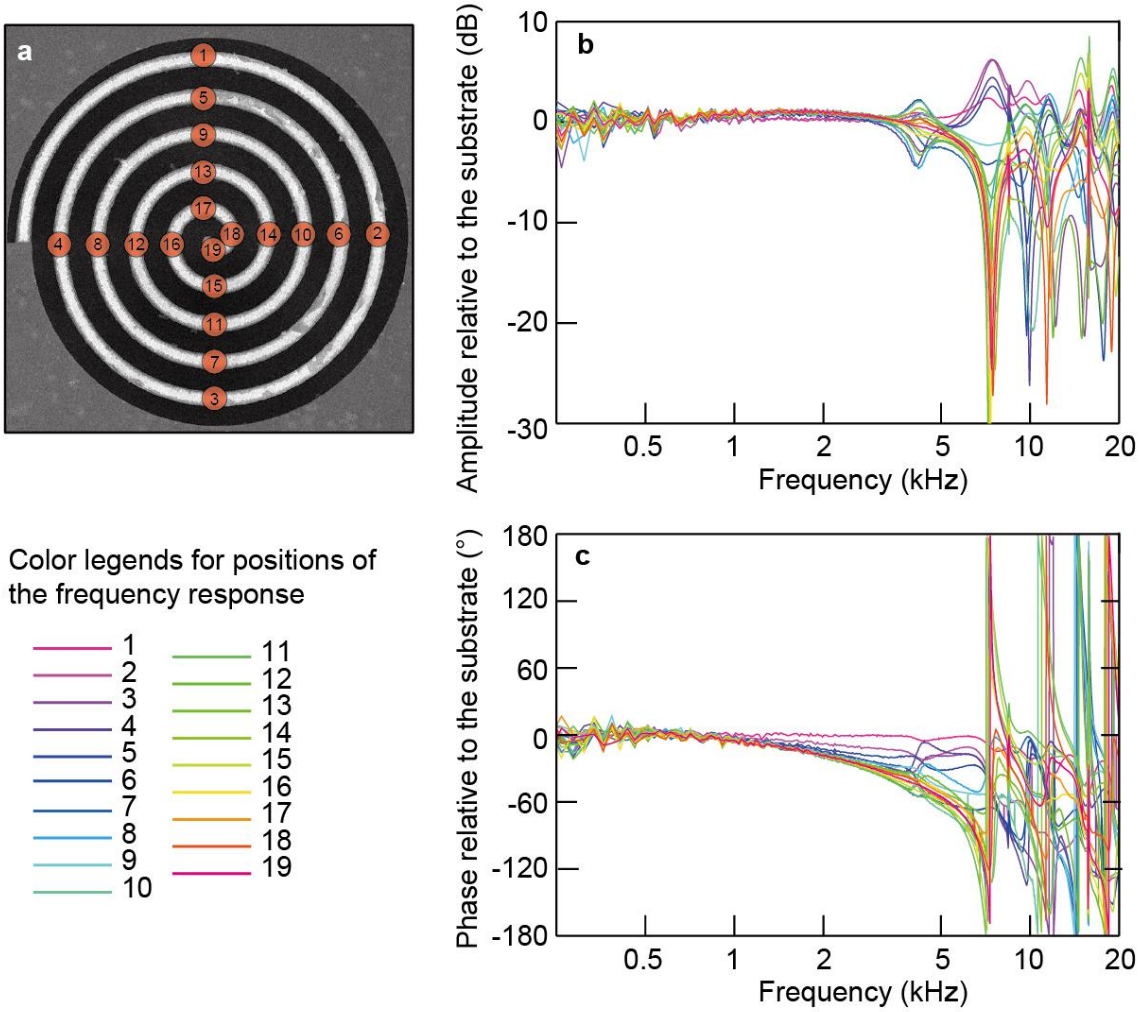

Supplementary Figure 2. Laser Doppler vibrometer measurements of the mechanical resonance properties of the spiral beam. (a) A spiral image that indicates the 19 positions at which measurements were performed. (b) Velocity amplitude of the spiral mechanical vibration relative to the substrate. (c) Phase relative to the substrate.

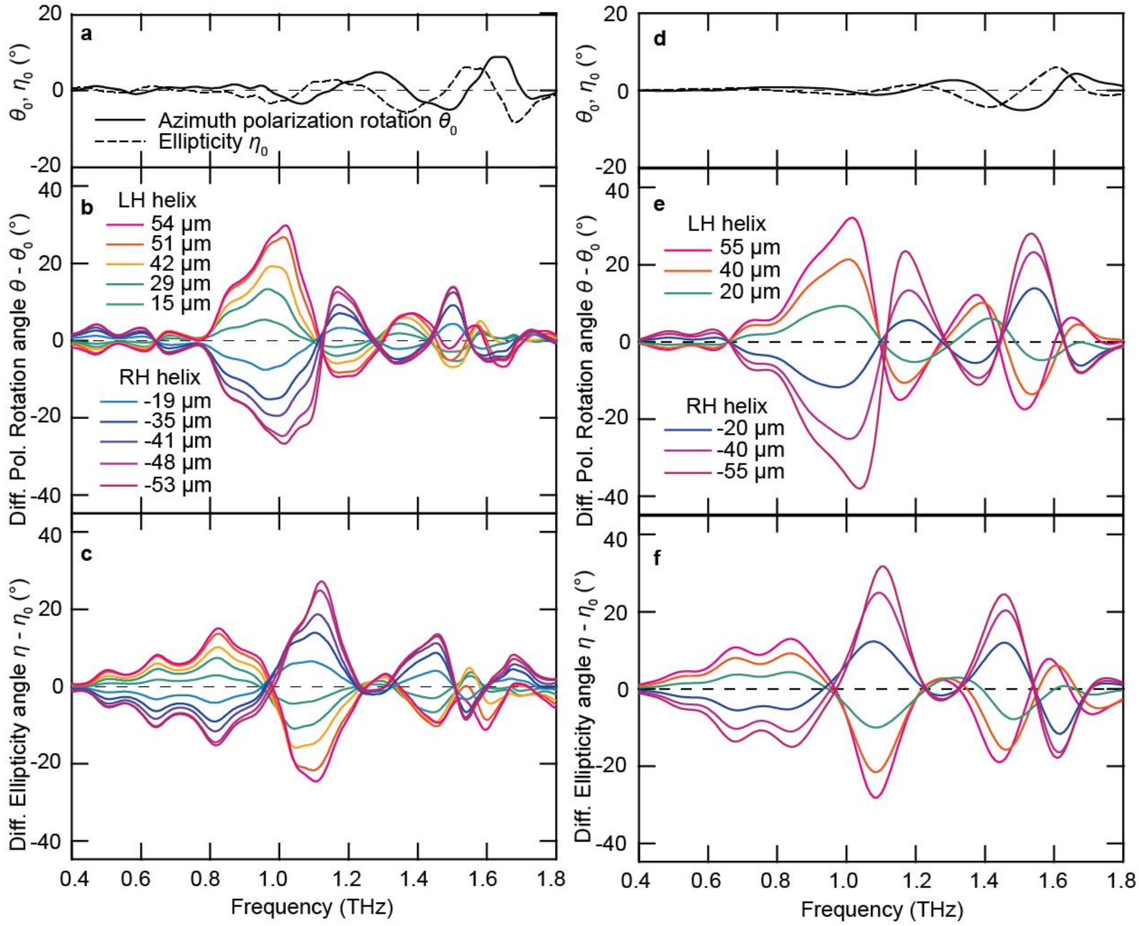

Supplementary Figure 3. Calculated optical activity spectra convolved by a Gaussian function to simulate the experimental measurement procedure. The spectral data before convolution were obtained by FEM calculations and correspond to those shown in Figure 3(f) through (h). Figure (a) through (c), which are identical to Figure 3(c) through 3(e) in the main article, are shown for comparison with the convolved plots. (d) Convolved polarization azimuth rotation θ_0 and ellipticity η_0 angles for the condition of no applied pressure. (e) The convolved azimuth polarization rotation angle $\theta - \theta_0$ spectrum for six deformation conditions. (f) The calculated differential ellipticity angle $\eta - \eta_0$ spectrum for six deformation conditions.

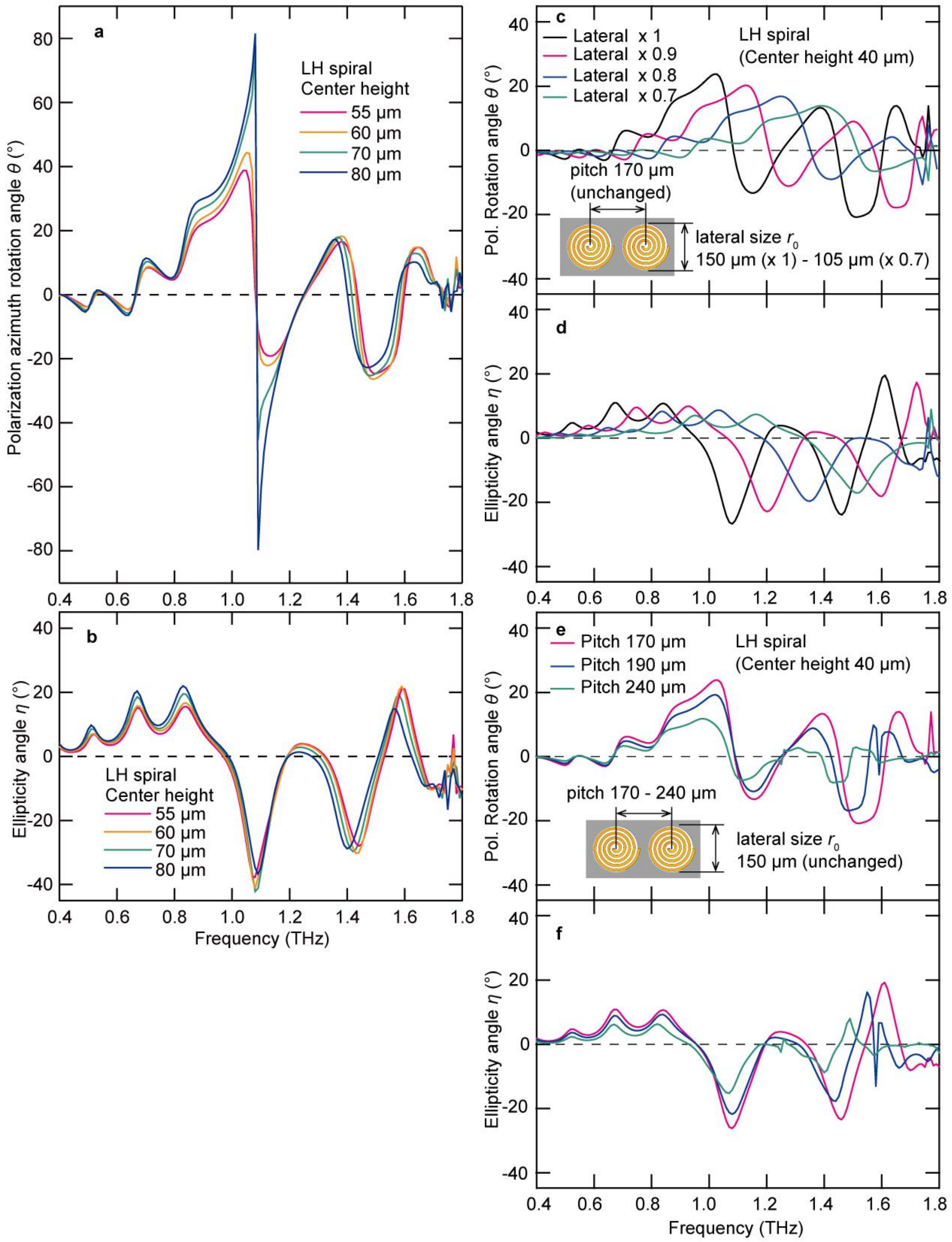

Supplementary Figure 4. Polarization spectra obtained through FEM calculations. (a), (b) Polarization azimuth rotation and ellipticity angular spectra for the spirals with different central heights. (c), (d) Polarization azimuth rotation and ellipticity angular spectra for laterally size-reduced spirals. (e), (f) Polarization azimuth rotation and ellipticity angular spectra for spirals with three different pitches.

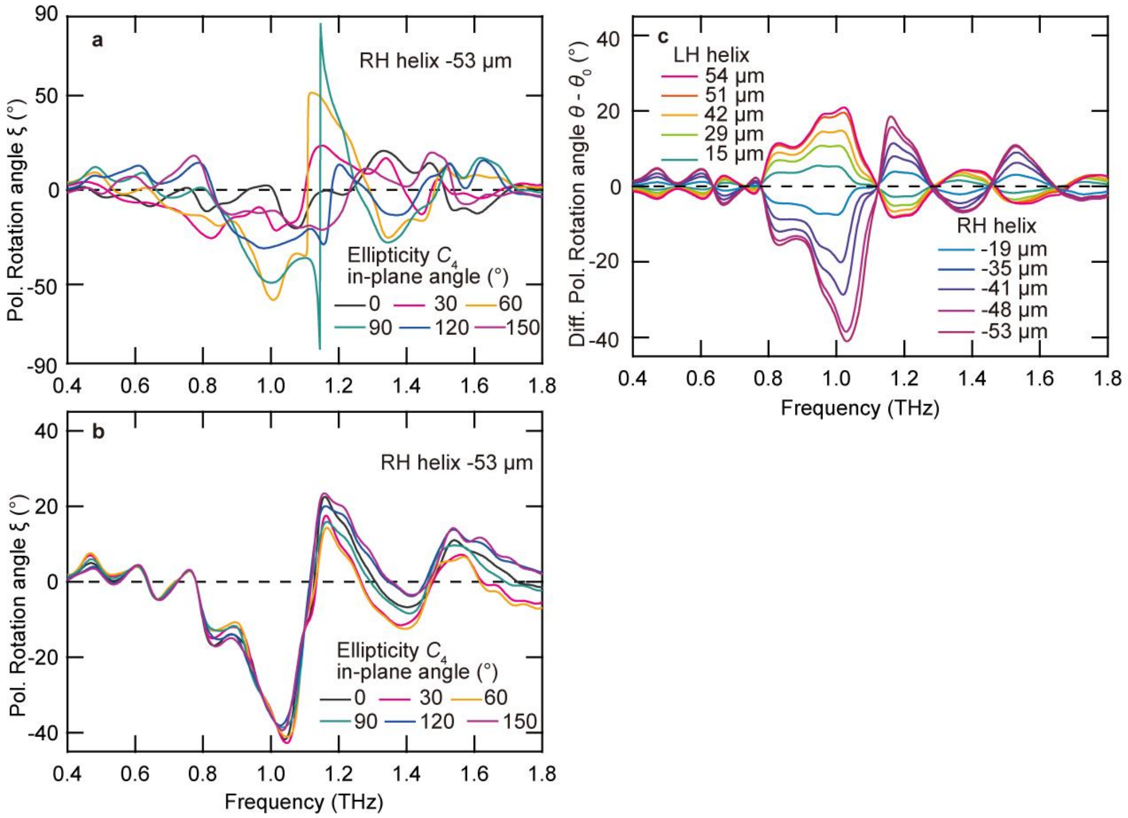

Supplementary Figure 5. Elimination of birefringence by arraying the spirals in a configuration with C_4 symmetry. (a) Spectra of the polarization azimuth rotation angle ξ without averaging over the in-plane angle for the C_1 metamaterial for six in-plane angles. All ellipticity spectra correspond to spirals with a center deformed height of –53 µm. For the derivation procedure, see the Equations in the Supplementary Information. (b) Spectra of the polarization azimuth rotation angle ξ without averaging over the in-plane angle for the C_4 device for six in-plane angles; the spirals have a center deformed height of –53 µm. (c) Differential polarization azimuth rotation angles $\theta – \theta_0$ of the C_4 spiral metamaterial.

Supplementary Note 1.

Actual three-dimensional shape of the deformed spiral

Photographs of the spiral in the pre- and on-actuation states are shown in Supplementary Figure 1(a) and 1(b), respectively. The pneumatic pressure is supplied from the underside of the spirals, thereby making left-handed three-dimensional spirals. The design parameters of the spirals are slightly different from those of the spiral metamaterials presented in the main manuscript in several respects: the arraying pitch is 190 µm, and no Au layer is coated on the spiral beam. However, the actuation characteristics are almost same. These photographs are extracted from a movie, which is also provided as supplementary information. The out-of-plane deformation profiles are plotted with respect to the angular position θ of the spiral beam (Supplementary Figure 1(c)). These profiles were obtained from a spiral of which design was the same with the spiral actually used in THz-TDS measurements in the main manuscript. The definition of θ is given in the inset of Supplementary Figure 1(c). Interpolation between data points was performed using a 3rd-order polynomial. A movie of dynamically actuated spirals is also provided as supplementary information. Almost-symmetrical deformation is observed in the left-handed (LH) and right-handed (RH) spiral profiles. These profiles are also used to construct a simulation model as described in the Methods section in the main manuscript.

Supplementary Note 2.

Laser Doppler analysis of the mechanical frequency response of the spiral beam

The mechanical resonance of the spiral beam with respect to the out-of-plane direction was measured using a laser Doppler vibrometer (MLD-221D, Neoarc Corp., Japan). To investigate the mechanical frequency response of a spiral structure alone, the endpoint of the spiral beams were fixed to the 300-µm-thick rigid Si substrate, unlike the spiral metamaterials used in the main manuscript, for which the spirals were fabricated on a 300-nm-thick Si membrane. The spiral metamaterial chip was mounted on a PZT actuator such that an external vibration in the out-of-plane direction could be applied. The laser spot was focused on the spiral beam to measure the vibrational velocity of the beam for frequencies from 250 Hz to 20 kHz. The vibrational velocity amplitude and phase of the spiral beam were obtained for 19 points of the spiral beam (Supplementary Figure 2(a)). The obtained vibrational velocity amplitude and phase plots are shown in Supplementary Figure 2(b) and 2(c), respectively. The vibrational velocity amplitude is divided by that of the substrate where spirals are not formed to eliminate artifacts from substrate vibration. The plotted values are thus the velocity amplitude relative to that of the substrate expressed in dB. The phase is also plotted as a value relative to that of the substrate. The vibrational velocity amplitude was flat from 250 Hz to 3 kHz, and no significant phase change was observed. At greater than 4 kHz, the vibrational velocity amplitude started to fluctuate, and an abrupt change in the phase appeared at approximately 7 kHz,

thus indicating the existence of a mechanical resonance of the spiral. This result indicates that the spiral was able to follow the external force actuation below 3 kHz, thereby assuring frequency modulation at a few kHz.

Supplementary Note 3.

Derivation of the Jones matrix and polarization effect from experimental data

We define the Jones matrix of a sample as

$$T = \begin{bmatrix} t_{xx} & t_{xy} \\ t_{yx} & t_{yy} \end{bmatrix}. \quad (1)$$

The electric-field vector $\mathbf{E}_s^1$ of a THz wave that passes through the sample can be written as

$$\mathbf{E}_s^1 = T\mathbf{E}_r, \quad (2)$$

where $\mathbf{E}_r$ is the electric-field vector of the reference THz wave measured without samples. In the same manner, the electric-field vector $\mathbf{E}_s^2$, which is that of a THz wave that passes through the sample rotated by an angle α in the plane normal to the propagation direction of the THz wave, can be written as

$$\mathbf{E}_s^2 = R(\alpha)TR(-\alpha)\mathbf{E}_r, \quad (3)$$

where $R(\alpha)$ is the rotation matrix for an angle of α,

$$R(\alpha) = \begin{bmatrix} \cos\alpha & -\sin\alpha \\ \sin\alpha & \cos\alpha \end{bmatrix} \quad (4)$$

Therefore, T can be calculated by

$$T = [\mathbf{E}_s^1\ \mathbf{E}_s^{2+}][\mathbf{E}_r\ \mathbf{E}_r^+]^{-1}, \quad (5)$$

where $\mathbf{E}_r^+$ and $\mathbf{E}_s^{2+}$ are defined as

$$\begin{aligned} \mathbf{E}_r^+ &= R(-\alpha)\mathbf{E}_r \\ \mathbf{E}_s^{2+} &= R(-\alpha)\mathbf{E}_s^2. \end{aligned} \quad (6)$$

We set α to 90° in the experiment.

Here, we assume that the direction of the in-plane optical axis of the sample is ϕ, whose definition is given in Figure 5(a). In this case, under rotation by $-\phi$, the Jones matrix T is converted to the following form:

$$R(-\phi)TR(\phi) = \begin{bmatrix} t_1 + t_3 & t_2 \\ -t_2 & t_1 - t_3 \end{bmatrix} \tag{7}$$

The off-diagonal term t_2 represents the optical activity, and an imbalance of the diagonal term t_3 represents anisotropy of the optical response, *i.e.*, birefringence.

To eliminate the effect of anisotropy and focus on the effect of optical activity, the Jones matrix T should be averaged over in-plane rotation. The Jones matrix T under in-plane rotation by α can be calculated using Equation (7) and is the following:

$$\begin{aligned} R(\alpha)TR(\alpha) &= R(\alpha)R(\phi)\begin{bmatrix} t_1 + t_3 & t_2 \\ -t_2 & t_1 - t_3 \end{bmatrix} R(-\phi)R(-\alpha) \\ &= \begin{bmatrix} t_1 + t_3 \cos 2(\alpha + \phi) & t_2 + t_3 \sin 2(\alpha + \phi) \\ -t_2 + t_3 \sin 2(\alpha + \phi) & t_1 - t_3 \cos 2(\alpha + \phi) \end{bmatrix}. \end{aligned} \tag{8}$$

Therefore, the Jones matrix T_{avg}, which is the matrix T averaged over in-plane rotation, is calculated using

$$T_{\mathrm{avg}} = \frac{1}{2\pi}\int_0^{2\pi} R(\alpha)TR(-\alpha)d\alpha = \begin{bmatrix} t_1 & t_2 \\ -t_2 & t_1 \end{bmatrix} \tag{9}$$

This Jones matrix corresponds to the general expression for an isotropic chiral medium in which the t_3 terms, which are related to birefringence, vanish.

By comparing Equation (1) and Equation (7), the following relation can be derived:

$$\begin{aligned} t_1 &= \frac{t_{xx} + t_{yy}}{2} \\ t_2 &= \frac{t_{xy} - t_{yx}}{2} \\ t_3 &= \sqrt{\left(\frac{t_{xx} - t_{yy}}{2}\right)^2 + \left(\frac{t_{xy} + t_{yx}}{2}\right)^2} \\ \phi &= \frac{1}{2}\tan^{-1}\left(\frac{t_{xy} + t_{yx}}{t_{xx} - t_{yy}}\right) \end{aligned} \tag{10}$$

.

Therefore, the parameters in Equation (7) can be calculated using the experimentally obtained data.

The chirality-induced polarization azimuth rotation angle θ and ellipticity angle η can be defined as

$$\begin{aligned} \theta &= \frac{1}{2}\tan^{-1}\left(\frac{S_2}{S_1}\right) \\ \eta &= \frac{1}{2}\sin^{-1}\left(\frac{S_3}{S_0}\right) \end{aligned} \tag{11}$$

where S_0, S_1, S_2, S_3 are the Stokes parameters, which are defined as

$$\begin{aligned} S_0 &= \tilde{E}_x \tilde{E}_x^* + \tilde{E}_y \tilde{E}_y^* \\ S_1 &= \tilde{E}_x \tilde{E}_x^* - \tilde{E}_y \tilde{E}_y^* \\ S_2 &= \tilde{E}_x \tilde{E}_y^* + \tilde{E}_y \tilde{E}_x^* \\ S_3 &= -i\left(\tilde{E}_x \tilde{E}_y^* - \tilde{E}_y \tilde{E}_x^*\right). \end{aligned} \tag{12}$$

The above description of the Stokes parameters follows the conventional descriptions in Supplementary Reference 1.

Here, we are interested in the polarization state in the case that $\mathbf{E}_\mathrm{r}$ is parallel to the *x*-axis. In this case, $(E_x, E_y) = (t_1, -t_2)$ and Equation (11), (12) are converted to

$$\begin{aligned} \theta &= -\frac{1}{2}\tan^{-1}\left(\frac{2\mathrm{Re}\{t_1^* t_2\}}{|t_1|^2 - |t_2|^2}\right) \\ \eta &= -\frac{1}{2}\sin^{-1}\left(\frac{2\mathrm{Im}\{t_1^* t_2\}}{|t_1|^2 + |t_2|^2}\right) \end{aligned} \tag{13}$$

.

The polarization azimuth rotation angle ξ and ellipticity angle ζ, including the effect of birefringence, can be calculated from Equation (8):

$$\begin{aligned} \xi &= -\frac{1}{2}\tan^{-1}\left(\frac{2\mathrm{Re}\{[t_1 + t_3\cos 2(\alpha+\phi)]^*[t_2 - t_3\sin 2(\alpha+\phi)]\}}{|t_1 + t_3\cos 2(\alpha+\phi)|^2 - |t_2 - t_3\sin 2(\alpha+\phi)|^2}\right) \\ \zeta &= -\frac{1}{2}\sin^{-1}\left(\frac{2\mathrm{Im}\{[t_1 + t_3\cos 2(\alpha+\phi)]^*[t_2 - t_3\sin 2(\alpha+\phi)]\}}{|t_1 + t_3\cos 2(\alpha+\phi)|^2 + |t_2 - t_3\sin 2(\alpha+\phi)|^2}\right) \end{aligned} \tag{14}$$

.

Supplementary Note 4.

Effect of the deconvolution processing of the calculated spectra

The frequency resolution of the experimentally obtained spectra was approximately 0.08 THz due to the limitation of the delay line length of the THz-TDS. The experimental spectra in Figure 3(a) through (e) are therefore blurred compared with the actual optical responses by this frequency resolution limit. By contrast, the calculated data did not suffer from such blurring, and thus the calculated data in Figure 3(f) through 3(h) are identical to the actual optical responses. To simulate the situation in which the calculated optical response is blurred by the experimental restriction on the frequency resolution, we plotted the calculated spectra convolved by a Gaussian function with σ = 0.08 THz in Supplementary Figure 3(d) through 3(f). The experimental data plots in Figure 3(c) through 3(e) in the main article are also presented in Supplementary Figure 3(a) through 3(c) for comparison. The convolved plots exhibited high consistency with the experimental data with respect to the amplitude of the optical activity and the spectral shapes. These results imply that the maximum amplitudes of the experimentally prepared metamaterial were underestimated by the limitation of the frequency resolution. Increasing the frequency resolution of the THz-TDS or employing a CW monochromatic THz light source may allow the correct value of the optical activity

of the metamaterial to be measured.

Supplementary Note 5.

Effects of the dimensions of the spiral metamaterial on the polarization spectra

The attainable maximum amplitudes of the optical activity were numerically investigated using commercial finite element method software (COMSOL ver. 4.4, COMSOL, USA). The LH spiral model with the original lateral size (r_0 = 150 µm) was used for the simulation. The optical activities for the spirals with four central heights, 55, 60, 70, and 80 µm, were calculated. These central heights are slightly higher than those used in the experiment. The pitch of the spirals was maintained at 170 µm. Plot legends are shown in Supplementary Figure 4(a). The polarization azimuth rotation angle spectra are shown in Supplementary Figure 4(a). The rotation angles exhibited an abrupt change around 1.1 THz for the 70- and 80-µm-height spirals exceeding 90°. This result indicates complete 90° polarization rotation is potentially possible with the proposed metamaterial. The ellipticity angle spectra are plotted in Supplementary Figure 4(b), and the maximum ellipticity angle of –42° was obtained for the 70-µm-height spiral with 170-µm pitch. The further increase in the pitch did not produce an increase in the ellipticity angle, and the maximum ellipticity angle for the 80-µm-height spirals decreased to –37°. Because the maximum of the ellipticity angle is ±45°, the proposed spiral can produce nearly circular polarization states from the linearly polarized incident light.

The effects of the size of the spiral on the polarization spectra were also numerically investigated. The lateral spiral size and arraying pitch of the spirals were adopted as the parameters that were used to tune the dimensions of the spiral model. First, the effect of the lateral spiral size was examined. An LH spiral model with a central height of 40 µm was used as a starting model; it was prepared following the procedure described in the Methods section of the main manuscript. Then, the lateral size of the model was reduced to 1 (identical to the starting model), 0.9, 0.8, and 0.7 times the lateral size of the starting model; these values correspond to the size reduction of r_0 of the numerical expression of Archimedean spiral described in the Methods section of the main manuscript and the beam width. The central height of the spiral and arraying pitch (170 µm) were kept unchanged. The polarization spectra and polarization azimuth rotation and ellipticity angles of these models are shown in Supplementary Figure 4(c) and 4(d), respectively. The lateral size had a significant influence on the resonant frequency. A decrease in the lateral size tended to shift the resonance frequency toward a higher frequency; thus, the lateral size is an important parameter for tuning the working frequency of the spiral metamaterial. In a second step, polarization spectra for three different arraying pitches, 170, 190, and 240 µm, were calculated (Supplementary Figure 4(e) and 4(f)). These spirals were also modeled with the procedure described in the Methods section of the

main manuscript, and the height of the center was set to 40 µm. Although there was a slight shift in the peak positions in the spectra over 1.4 THz, depending on the pitch, the primary resonance frequency of approximately 1 THz did not depend on the pitch. The pitch does not determine the working frequency, but it does have an effect on the optical density of the metamaterial. This result indicates that a densely arrayed metamaterial exhibits a high degree of optical activity.

Supplementary Note 6.

Effect of the in-plane angle for the polarization azimuth rotation angle spectra

The polarization azimuth rotation angle spectra for different in-plane angles of the metamaterial are shown in Supplementary Figure 5. The procedures used to obtain these spectra were identical to those described in the manuscript to obtain ellipticity angles (Figure 5). The polarization azimuth rotation angle spectra of the C_1 arrangement exhibited a large dependence on the in-plane angle (Supplementary Figure 5(a)). By contrast, the dependence on the in-plane angle was eliminated in the C_4 arrangement (Supplementary Figure 5(b)). The polarization azimuth rotation spectra of the C_4 metamaterial for different deformation conditions are shown in Supplementary Figure 5(c) to exhibit a similar spectral shape as those for the C_1 metamaterial in Figure 3.

Supplementary Reference

1 Born, M., Wolf, E. & Bhatia, A. B. *Principles of optics : electromagnetic theory of propagation, interference and diffraction of light*. (Cambridge University Press, 1999).
